# An Integrative Pharmacology-Based Analysis of Refined Qingkailing Injection Against Cerebral Ischemic Stroke: A Novel Combination of Baicalin, Geniposide, Cholic Acid, and Hyodeoxycholic Acid

**DOI:** 10.3389/fphar.2020.00519

**Published:** 2020-05-08

**Authors:** Chongyang Ma, Xueqian Wang, Tian Xu, Shuang Zhang, Shuling Liu, Changming Zhai, Zisong Wang, Jie Mu, Changxiang Li, Fafeng Cheng, Qingguo Wang

**Affiliations:** ^1^School of Traditional Chinese Medicine, Capital Medical University, Beijing, China; ^2^School of Traditional Chinese Medicine Department, Beijing University of Chinese Medicine, Beijing, China; ^3^Department of Liver Disease, Guangdong Province Hospital of Traditional Chinese Medicine Zhuhai Branch, Zhuhai, China; ^4^Department of Traditional Chinese Medicine, Beijing Chaoyang Hospital, Capital Medical University, Beijing, China

**Keywords:** network pharmacology, ischemic stroke, combination of drug, Chinese medicine, protein protein-interaction (PPI) network, topological analysis, Qingkailing, modern Chinese medicine

## Abstract

Stroke is the second leading cause of death after heart disease globally and cerebral ischemic stroke accounts for approximately 70% of all incident stroke cases. We selected four main compounds from a patent Chinese medicine, Qingkailing (QKL) injection, including baicalin from *Scutellaria baicalensis* Georgi (Huang Qin), geniposide from *Gardenia jasminoides* J. Ellis (Zhizi), and cholic acid and hyodeoxycholic acid from *Bovis Calculus* (Niuhuang) with a ratio of 4.4:0.4:3:2.6 m/m, to develop a more efficacious and safer modern Chinese medicine injection against ischemic stroke, refined QKL (RQKL). In this study, we investigated multiple targets, levels, and pathways of RQKL by using an integrative pharm\acology combining experimental validation approach. In silica study showed that RQKL may regulate PI3K-Akt, estrogen, neurotrophin, HIF-1, MAPK, Hippo, FoxO, TGF-beta, NOD-like receptor, apoptosis, NF-kappa B, Wnt, chemokine, TNF, Toll-like receptor signaling pathways against ischemic stroke. The experimental results showed that RQKL improved neurological function and prevented infract volume and blood-brain-barrier damage. RQKL inhibited microgliosis and astrogliosis, and protected neurons from ischemic/reperfusion injury. RQKL also inhibited cell apoptosis and affecting the ratio of the anti-apoptosis protein B-cell lymphoma-2 (Bcl2) and pro-apoptosis protein Bcl2-associated X protein (Bax). Western blot analysis showed that RQKL activated AKT/PI3K signaling pathway and antibody array showed RQKL inhibited inflammatory response and decreased proinflammatory factor Tnf, Il6, and Il1b, and chemokines Ccl2, Cxcl2, and Cxcl3, and increased anti-inflammatory cytokine Il10. In conclusion, RQKL protected tissue against ischemic stroke through multiple-target, multiple signals, and modulating multiple cell-types in brain. This study not only promoted our understanding of the role of RQKL against ischemic stroke, but also provided a pattern for the study of Chinese medicine combining pharmaceutical Informatics and system biology methods.

## Introduction

Stroke is the second leading cause of death after heart disease globally and is associated with the highest disability-adjusted life-years lost of any disease in China (Wu et al., 2019). Approximately 795,000 new stroke cases are reported in the US yearly and 4.3 times this number was recently surveyed by the nationwide community-based study, NESS-China (Wang W. et al., 2017). Cerebral ischemic stroke accounts for approximately 70% of all incident stroke cases. Since the 1990s, intravenous alteplase has been considered the only evidence-based therapeutic agent for improving the prognosis of patients with cerebral ischemic stroke and accepted as standard of care all around the world (Powers et al., 2019). However, its short therapeutic window and high risk of hemorrhagic complications have hampered its widespread adoption in China. Therefore, exploring novel therapeutic strategies to treat ischemic stroke is an urgent need.

Qingkailing (QKL) injection, is a patent traditional Chinese medicine formulation approved by the China Food and Drug Administration to treat ischemic stroke for over 30 years. We previously study screened a novel modern drug combination (refined Qingkailing, RQKL) consisting of four compounds, baicalin (CAS number 21967-41-9) from *Scutellaria baicalensis* Georgi (Huang Qin), geniposide (CAS number 24512-63-8) from *Gardenia jasminoides* J. Ellis (Zhizi), and cholic acid (CAS number 81-25-4) and hyodeoxycholic acid (CAS number 83-49-8) from *Bovis Calculus* (Niuhuang) with a ratio of 4.4:0.4:3:2.6 m/m. Our previous study showed that RQKL protected the brain against ischemia-reperfusion (I/R) injury *in vivo* and *in vitro* (Cheng et al., 2012; Cheng et al., 2018). However, the underlying mechanisms and core signaling pathways mediating the multi-linked and multi-targeted effects of RQKL against ischemic stroke are still unknown.

Generally, natural bioactive compounds exert therapeutic effects through multiple targets and pathways that cannot be accurately detected solely using conventional pharmacological approaches.

**Figure 1:**
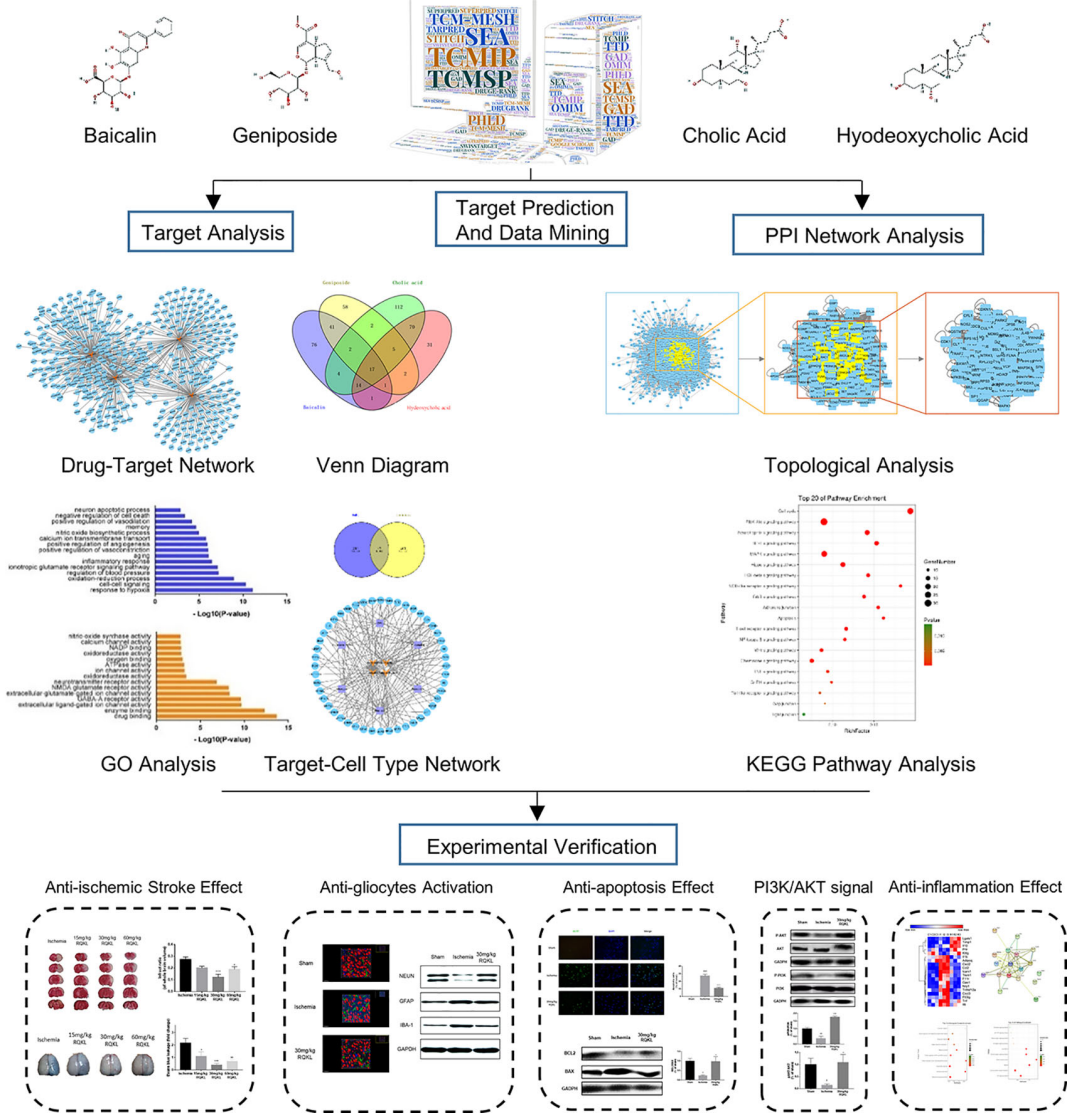
Schematic diagram of combining integrative pharmacology and experimental approach used in this work.

Integrative pharmacology could enhance the comprehension and facilitate the prediction of potential targets, pathways, and consequences, which might provide clues for designing subsequent research studies. In this work, we used an integrative pharmacology approach with the goal of understanding the systemic, organ-related, and molecular effects of RQKL. This approach combined the prediction of multiple drug targets, visualization of compound-target network and target-cell-type network, topological analysis of protein-protein interaction (PPI) networks and gene ontology (GO), and KEGG pathway analysis of core targets. Importantly, our experimental results largely validated the mechanism of action of RQKL, as predicted by the integrative pharmacology analysis (**Figure 1**).

## Materials and Methods

### Materials and Reagents

The RQKL used in this study was a mixture of baicalin, geniposide, cholic acid, and hyodeoxycholic acid (4.4:0.4:3:2.6). Baicalin (CAS number 21967-41-9), geniposide (CAS number 24512-63-8), and cholic acid (CAS number 81-25-4), and hyodeoxycholic acid (CAS number 83-49-8) were purchased from Shanghai Aladdin Biochemical Technology Co., Ltd. (Shanghai China). Protease inhibitor, radioimmunoprecipitation assay (RIPA) lysis buffer, and enhanced chemiluminescence (ECL) reagent were obtained from Applygen Technologies Inc. (Beijing China). The antibodies against B-cell lymphoma-2 (Bcl2, 12789-1-AP) and glyceraldehyde-3-phosphate dehydrogenase (GAPDH, 10494-1-AP) were obtained from Proteintech Group, Inc (Rosemont, USA). The antibodies against BCL2-associated X protein (BAX, #2772), serine-threonine protein kinase (AKT, #9272), phosphorylated-AKT (pAKT, #9271), phosphatidylinositol-4,5-Bisphosphate 3-kinase (PI3K, #4249), and phosphorylated-PI3K (pPI3K, #4228) were obtained from Cell Signaling Technology (Boston, USA). The antibodies against glial fibrillary acidic protein (GFAP, ab7260) was obtained from Abcam (Cambridge, USA). The antibodies against GFAP labeled Alexa Fluor 488 (MAB3402X) and neuronal nuclei antigen (NEUN, MAB37) were purchased form Millipore (Darmstadt, Germany) and the antibody against ionized calcium binding adaptor molecule-1 (IBA1, 019-19741) was purchased from WAKO CHEMICAL, CO., LTD. (Japan). Terminal deoxynucleotidyl transferase (TdT)-mediated deoxyuridine triphosphate (dUTP) nick-end labeling (TUNEL) apoptosis detection kit was purchased from Roche Applied Science (Mannheim, Germany). Rat cytokine array antibody arrays (GSR-CAA-67) were purchased from RayBiotech Life (California, USA).

### Construction of the Compound-Target and Disease-Target Databases

To identify the corresponding targets of the four active ingredients of RQKL, several approaches combined with a chemometric method, information integration, and data-mining were implemented. First, the biological targets of the active ingredients were obtained from SEA (http://sea.bkslab.org/) (Keiser et al., 2007), STITCH (http://stitch.embl.de/) (Szklarczyk et al., 2016), DrugE-Rank (http://datamining-iip.fudan.edu.cn/service/DrugE-Rank) (Yuan et al., 2016), PhlD (http://phid.ditad.org/) (Hu et al., 2014), SuperPred (http://prediction.charite.de/index.php?site=home) (Nickel et al., 2014), SwissTarget (http://www.swisstargetprediction.ch/) (Gfeller et al., 2014), and TarPred (http://www.dddc.ac.cn/tarpred) (Liu et al., 2015). All active compounds were also sent to TCMIP (http://www.tcmip.cn/TCMIP/index.php/Home/Index/index.html) (Xu et al., 2019) TCM-Mesh (http://mesh.tcm.microbioinformatics.org/) (Zhang et al., 2017), TCMSP (http://tcmspw.com/tcmsp.php) (Ru et al., 2014), and Google Scholar to mine compound-target interactions. Please see detailed information in **Supplementary Table S1**.

Known therapeutic targets for ischemic stroke were collected from the DrugBank (http://www.drugbank.ca/) (Wishart et al., 2018), Online Mendelian Inheritance in Man (OMIM) (http://www.omim.org) (Hamosh et al., 2005), Genetic Association (GAD, http://geneticassociationdb.nih.gov/) (Becker et al., 2004), and Therapeutic Target Database (TTD, https://db.idrblab.org/ttd/) (Yang et al., 2016) databases. After deleting the redundant information, 321 known therapeutic targets for the treatment of ischemic stroke were included in this study. Please see detailed information in **Supplementary Table S2**.

### Screening of Target Related Cell-Type

AlzData (http://www.alzdata.org/) database contains gene expression data of different cell-types from human brain single cell RNA-seq (GSE67835) and was used to recognize target related cell-type in the present study (Xu et al., 2018). In briefly, after overlapping compound-target and disease-target databases, we input the obtained targets into AlzData database to investigate each target related cell-types.

### Network Construction

Two kinds of networks in this study were established using Cytoscape (version 3.2.1) software (Shannon et al., 2003): compound-target network and target-cell-type network. Compound-target network was composed of compounds and their potential targets, which was built to reveal the compound-target interactions. Target-cell-type network was built based on the potential targets and their related cell-types.

### Protein-Protein Interaction Network Construction

PPI data were imported from six currently available PPI databases, including the Biological General Repository for Interaction Datasets (BioGRID), Biomolecular Interaction Network Database (BIND), Molecular INTeraction Database (MINT), Human Protein Reference Database (HPRD), and Database of Interacting Proteins (DIP), which were searched using BisoGenet, a Cytoscape plugin (Martin et al., 2010). An interactive network for the candidate drug targets and known ischemic stroke-related targets of RQKL was constructed based on their interaction data and was visualized using the Cytoscape software.

### Definition of Topological Feature Set for the Network

As previously mentioned the topological properties of every node in the interaction network were analyzed by calculating six measures: “betweenness centrality (BC),” “degree centrality (DC),” “eigenvector centrality (EC),” “closeness centrality (CC),” “network centrality (NC),” and “local average connectivity (LAC)” using CytoNCA (Wang X. et al., 2017). The definitions and computation equations of these six parameters represent the topological importance of a node in the network. More important nodes receive higher quantitative values within the network than less important nodes did (Tang et al., 2015).

### Gene Oncology Enrichment and Pathway Analysis

Further, we performed GO analysis of the 438 non-repetitive putative targets of RQKL using the Database for Annotation, Visualization, and Integrated Discovery (DAVID) to gain insights into their involvement in two different categories namely, biological processes (BP) and molecular function (MF) (Sherman and Lempicki, 2009). Tissue enrichment analysis was performed using FunRich software (http://www.funrich.org) (Pathan et al., 2015). Then, we performed Kyoto Encyclopedia of Genes and Genomes (KEGG) signaling pathway enrichment analysis of the 189 candidate targets of RQKL after the topological analysis. A P < 0.05 was considered significant, and the enriched GO terms were identified using the hypergeometric test. A Bubble Chart was plotted using the OmicShare tools, a free online platform for data analysis (www.omicshare.com/tools).

### Animals

Adult male Sprague-Dawley rats (220–230 g), provided by Vital River Laboratory Animal Technology (number SCXK 2016-0006, Beijing, China), were housed in the laboratory animal room and maintained at 25 ± 1°C with 65 ± 5% humidity on a 12-h light/dark cycle (lights on from 07:30 to 19:30) for at least 1 week before the experiments. Animals were provided food and water *ad libitum*. The animal experimental design and protocols used in this study were approved by the Ethics Review Committee for Animal Experimentation of Beijing University of Chinese Medicine (BUCM-4-2017090116-3016).

### Transient Middle Cerebral Artery Occlusion

All animals were fasted overnight but allowed free access to water and were then randomly assigned to five groups: low-, medium-, and high-dose RQKL treatment; ischemic, and control groups. The transient middle cerebral artery (tMCA) occlusion (tMCAO) model was established as described previously to induce focal cerebral I/R injury (Zheng et al., 2014). The right MCA was occluded using a poly-L-lysine-coated nylon suture, which was inserted from the external carotid artery into the common carotid artery, and after a 90-min occlusion, the MCA-suture was carefully removed for reperfusion. The body temperature of the rats was maintained at 37°C. The sham group was subjected to the same procedure except for the nylon thread insertion. Rats in the low-, medium, and high-dose RQKL groups were administered intraperitoneal injections of RQKL dissolved in saline water at doses of 15, 30, and 60 mg/kg, respectively, and those in the ischemic group were administered physiological saline at the same volume. The first injection was performed immediately after model establishment, followed by administration after 4 h, and once every 12 h after that. At 24 h after reperfusion, 10 rats in each group were euthanized, and the brains were rapidly excised for histomorphological assay or protein detection.

### Neurological Assessment

The neurological deficit score of each rat was measured 24 h after tMCAO induction in a blinded fashion according to a well-established five-point neurological scale (Longa et al., 1989): 0 = no apparent deficits, 1 = failure to fully extend the right forepaw, 2 = circling to the right, 3 = falling or leaning over to the right, 4 = no spontaneous walking and a depressed level of consciousness, and 5 = death.

### Infarct Volume Assessment

Following neurological function evaluation, the rats were euthanized, and the brains were harvested for triphenyltetrazolium chloride (TTC) staining. The percentage infarct volume relative to the entire brain represented the degree of cerebral infarction. Serial coronal sections (2 mm thick) were prepared and soaked in 2% TTC phosphate buffer at 37°C for 10 min in the dark. Normal brain tissues were stained red while infarct tissues were not stained (white). The sections were soaked in 4% paraformaldehyde (PFA) phosphate buffer for 30 min, arranged in order and scanned (Tsinghua Unisplendour A688, Xi’an, China). Areas of red and white staining were measured using a computer color multimedia image analysis system (Image-Pro Plus 6.0, Media Cybernetics, WY, USA). The percentage of the infarction was calculated using the following equation: infarct volume (%) = infarct volume/total volume of slice × 100.

### Blood-Brain-Barrier Permeability

The blood-brain barrier (BBB) permeability was determined using Evans blue (EB) extravasation 24 h after tMCAO. In brief, EB dye (2%, 4 ml/kg) was injected over a 2-min period into the left femoral vein at a dose of 2 ml/kg and allowed to circulate for 1 h. Rats were anesthetized and perfused transcardially using saline to remove the intravascular EB dye. After decapitated, the entire brain of each animal was removed, homogenized in physiological phosphate-buffered saline (PBS), trichloroacetic acid was added to precipitate the protein, and then the tissue homogenates were cooled and centrifuged. The EB absorbance of the resulting supernatant was measured at 620 nm using a spectrophotometer.

### Nissl Staining

Coronal brain sections from four equidistant brain levels, 1 mm apart, were stained with cresyl violet according to a standard protocol. For Nissl staining, air-dried sections were fixed in 4% PFA solution for 15 min; immersed in 100, 95, 85, and 70% ethanol, followed by double distilled water for 3 min each; stained for 15 min with filtered cresyl violet solution [Sigma, 0.2% (w/v)]); and then briefly rinsed in double distilled water. Finally, the sections were dehydrated again in 70, 95, and 100% ethanol for 1 min each; placed in xylene for another 10 min; and then coverslipped. Nissl staining was performed to examine the neuronal injury, and necrotic neurons (red triangles) showed the absence of Nissl’s bodies in the cytoplasm, a shrunken intercellular space, and deep staining. The following four scores were used to evaluate necrotic neurons in the infarct area: 0, normal; 1, damaged neurons were < 25%; 2, damaged neurons were 25–50%; 3, damaged neurons were 50–75%; and 4, damaged neurons were > 75%(Sun et al., 2009).

### Terminal Deoxynucleotidyl Transferase-Mediated Deoxyuridine Triphosphate Nick-End Labeling Staining

Ischemic stroke-induced DNA fragmentation was quantified in frozen sections using the TdT-mediated dUTP nick-end labeling (TUNEL) assay according to the manufacturer’s instructions. Briefly, air-dried sections were fixed with methanol-free 4% PFA at room temperature for 15 min and washed twice with PBS. The sections were immersed in equilibration buffer for 10 min, which was then replaced by a mixture of 1 μl TdT enzyme, 5 μl nucleotide mix, and 45 μl equilibration buffer. The sections were then kept at 37°C for 90 min, and then SSC (2×) was added for 15 min at room temperature to terminate the TdT enzyme reaction.

### Confocal Microscopy and Morphology Analysis

Brains were cut into coronal sections (40 μm) on a cryostat, placed in 1 ml of anti-freeze solution (40% PBS, 30% ethylene-glycol, 30%, glycerol, v/v) and stored at −20°C until immunohistochemistry. Triple immunostaining was performed on coronal slices with the free-floating method as previously described (Lana et al., 2014). The following primary antibodies were used a mouse monoclonal anti-neuronal nuclei (NeuN, 1:200; Millipore, Billerica, MA) for neurons; a rabbit polyclonal anti-glial fibrillary acidic protein (GFAP, 1:1,000; DakoCytomation, Glostrup, Denmark) for astrocytes; a rabbit polyclonal anti-IBA1 (1:300, WAKO Pure Chem. Ind., Osaka, Japan) for microglia. Slices were observed under a ZEISS LSM 5 confocal laser scanning microscope. Confocal scans were taken at 0.5 μm z-steps keeping pinhole, contrast, and brightness constant. Semi-automated image analysis was performed using Bitplane IMARIS 7.4 3D image analysis software (Oxford Instruments, Concord, MA) and the 3-D reconstructed images of microglia and astrocyte were using surface function based on previously described (Radford et al., 2015).

### Western Blotting

The cortical tissue in the penumbra was collected for western blot analysis. The protein was extracted using RIPA lysis buffer containing protease inhibitor, and the concentration was measured using a bicinchoninic acid (BCA) protein assay kit (#CW0014, CWBio, China). Protein samples (50 µg) were separated on sodium dodecyl sulfate (SDS)-polyacrylamide gels and transferred onto a polyvinylidene fluoride membrane (Millipore Corporation, Billerica, MA, USA). The membrane was blocked with 5% nonfat dry milk in Tris-buffered saline containing 0.05% Tween-20 (TBST) buffer and then incubated with primary antibodies against BCL2, BAX, pAKT, AKT, pPI3K, PI3K, NEUN, GFAP, IBA-1, and GAPDH overnight at 4°C. Subsequently, the membranes were incubated for 1 h at room temperature with secondary antibodies coupled to horseradish peroxidase (1: 10,000). The antigen-antibody complexes were then exposed to the ECL reagent and visualized using a c600 western blot imaging system (Azure Biosystems, Dublin, CA, USA). The protein levels of pro-inflammatory mediators were expressed as relative integrated intensity and were normalized to that of GAPDH.

### Cytokine Antibody Microarray

Rat cytokine array antibody arrays were used to analyze cytokine expression profiles in brain samples according to the manufacturer's instructions. A digital imaging system (InnoScan 300 Microarray Scanner, Innopsys) was used to detect chemiluminescent signals which were analyzed using ImageJ software (NIH). Measured cytokines included the following: ADIPOQ, AGER, CCL11, CCL2, CCL27, CCL3, CCL5, CD48, CD80, CD86| CDH3, CNTF, CSF2, CX3CL1, CXCL1, CXCL2, CXCL3, CXCL5, DCN, EPHA5, EPO, F11R, FGFBP1, FLT3LG, GAS1, GFRA1, HAVCR1, HGF, ICAM1, IFNG, IGDCC4, IL10, IL13, IL17F, IL1A, IL1B, IL1RL2, IL1RN, IL2, IL22, IL2RA, IL3, IL4, IL6, IL6ST, IL7, INHBA, KITLG, LGALS1, LGALS3, NGF, NOTCH1, NOTCH2, NRP1, NRP2, PDGFA, PPBP, PRL, PRLR, SELL, TIMP1, TIMP2, TNF, TNFRSF9, TNFSF12, TNFSF9, TREM1, VEGFA.

### Statistical Analysis

Statistical analysis was performed using the GraphPad Prism 5 software. Normally distributed data and homogeneous variances were expressed as means ± standard error of the mean (SEM). These data were analyzed by one-way analysis of variance (ANOVA) followed by a *post hoc* Bonferroni multiple comparison test or Dunnett's test. Nonnormally distributed data were expressed as the median. Kruskal–Wallis test was used for the within-group differences comparation, and the Wilcoxon rank test was used to compare two groups. A P < 0.05 was considered significant.

## Results

### Network Pharmacological Analysis of Refined Qingkailing

#### Construction and Analysis of Compound-Target Network of Refined Qingkailing

**Figure 2:**
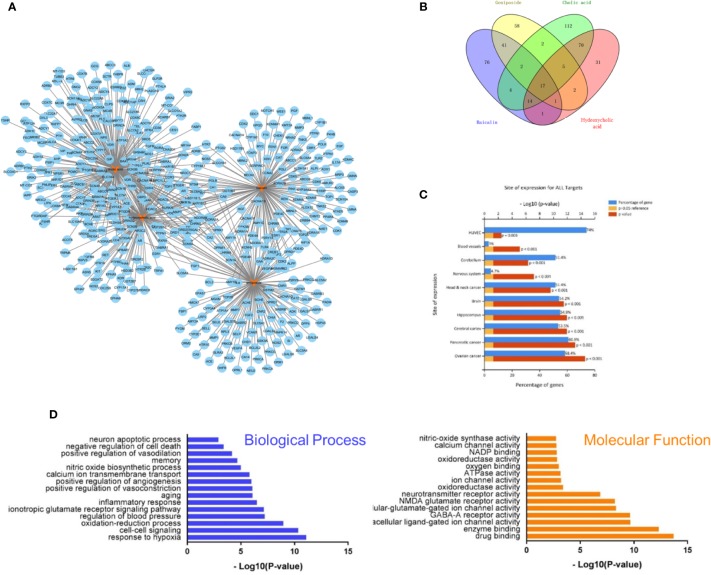
Compound-target interaction network and preliminary gene ontology (GO) analysis of drug targets. **(A)** Compound-target network of refined Qingkailing (RQKL). **(B)** Preliminarily screening targets of four compounds in RQKL. **(C)** Tissue enrichment analysis of drug target **(D)** GO analysis of drug targets classified into two categories, biological process (BP), and molecular function (MF).

RQKL is composed of baicalin, geniposide, cholic acid, and hyodeoxycholic acid, which have all been identified to be crucial bioactive natural compounds of a famous patent Chinese medicine, QKL injection. In this work, we explored the therapeutic targets of RQKL predicted using multiple online databases as previously described. Then, a network of potential targets of the compounds in RQKL was constructed using Cytoscape software as shown in **Figure 2A**, and 652 compound-target interactions were generated between the four compounds and 438 non-repetitive targets: 156, 129, 226, and 141 for baicalin, geniposide, cholic acid, and hyodeoxycholic acid, respectively. As shown in **Figure 2B**, we found that 16 differential genes were overlapping among all the targets of each compound, indicating a synergistic effect of RQKL compounds on these targets, such as the dopamine receptor D2 (DRD2) and estrogen receptor 1 (ESR1), adenosine A1 receptor (ADORA1), and retinoid X receptor alpha (RXRA).

#### Tissue and Gene Ontology Enrichment of Refined Qingkailing Targets

Tissue enrichment using the FunRich software (http://www.funrich.org) showed in **Figure 2C**, indicated that all targets of the four compounds were located in the cerebral cortex, hippocampus, and blood vessels, especially human umbilical vein endothelial cells (HUVEC), indicating a therapeutic effect on brain disease. GO analysis of the putative RQKL targets described based on BP and MF terms were constructed using the DAVID database. In total, 597 BPs and 183 MFs that were enriched for this dataset were identified, which consisted of 449 BPs and 145 MFs with P <0.05. **Figure 2D** illustrates an overview of the GO analysis with 15 remarkably enriched terms in the BP and MF categories. We noticed that some BPs and MFs might be associated with the pathogenesis of ischemic stroke such as response to hypoxia, inflammatory response, negative regulation of cell death, and calcium channel activity, indicating the potential mechanisms of action of RQKL in ischemic stroke.

### Construction and Analysis of Compound-Target-Cell Type Network of Refined Qingkailing Against Ischemic Stroke

**Figure 3:**
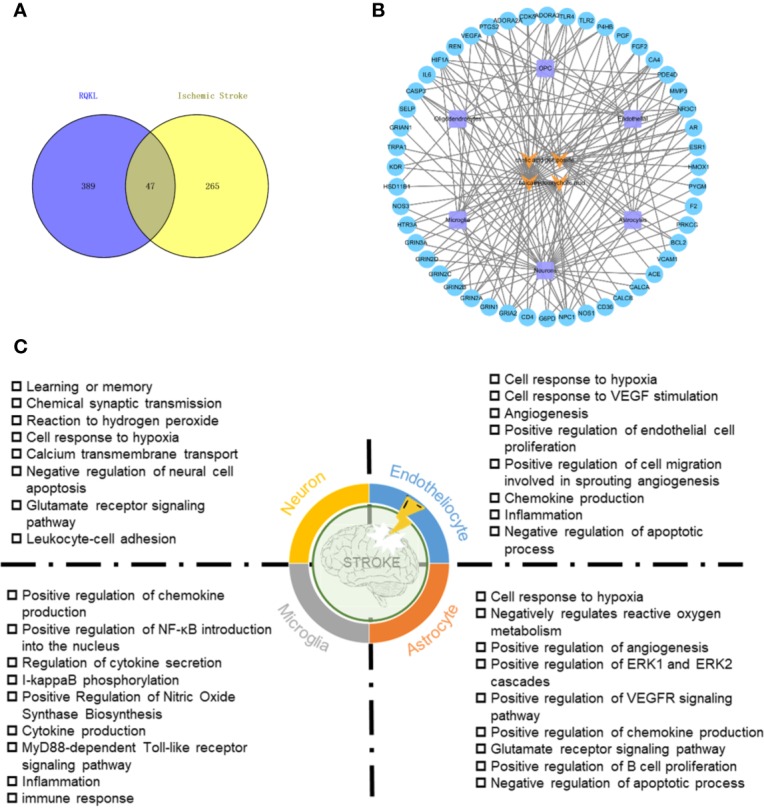
Ischemic stroke-related targets of refined Qingkailing (RQKL) regulated multiple cell type *via* multiple biological processes. **(A)** Veen diagram of compound targets of RQKL and ischemic stroke-related targets. **(B)** The compound-target-cell-type network of RQKL anti-ischemic stroke effect. The yellow refers to the compounds of RQKL. The purple represents six cell-types in brain and the blue represents intersectant targets of RQKL and ischemic stroke. **(C)** Biological process enrichment analysis of targets related to different cell-types in brain.

To evaluate targets of RQKL against ischemic stroke, we collected 321 ischemic stroke-related targets from four existing databases, namely the DrugBank, Online Mendelian Inheritance in Man, Genetic Association, and Therapeutic Target database. A Venn diagram was shown in **Figure 3A** and 47 targets were found overlapped between compound targets and ischemic stroke targets. AlzData online database was used to understand the relationship between these 47 targets and cell types in brain. A compound-target-cell type network was constructed in **Figure 3B**, and we found 31 targets were related to neurons, 14 targets were related to astrocytes, 13 targets were related to endothelial cells, and 11 targets were related to microglia, indicating an effect of RQKL on these cells. BP enrichment showed in **Figure 3C** indicated different effects of RQKL on different cell type. In neuron, RQKL may involve in learning or memory, chemical synaptic transmission, response to hydrogen peroxide, cellular response to hypoxia, calcium transmembrane transport, negative regulation of neural cell apoptosis, and glutamate receptor signaling pathway. In astrocyte, cell response to hypoxia, negatively regulates reactive oxygen metabolism, positive regulation of angiogenesis, positive regulation of ERK1 and ERK2 cascades, positive regulation of VEGFR signaling pathway, positive regulation of chemokine production, glutamate receptor signaling pathway, positive regulation of B cell proliferation, and negative regulation of apoptotic process. In endotheliocyte, cell response to hypoxia, cell response to VEGF stimulation, angiogenesis, positive regulation of endothelial cell proliferation, positive regulation of cell migration involved in sprouting angiogenesis, chemokine production, inflammation, and negative regulation of apoptotic process. In microglia, RQKL may involve in some inflammation process, including positive regulation of chemokine production, positive regulation of NF-κB introduction into the nucleus, regulation of cytokine secretion, I-kappaB phosphorylation, positive regulation of nitric oxide synthase biosynthesis, cytokine production, MyD88-dependent Toll-like receptor signaling pathway, inflammation, and immune response.

### Identification of Core Anti-Ischemic Stroke Targets *via* Protein-Protein Interaction Network

**Figure 4:**
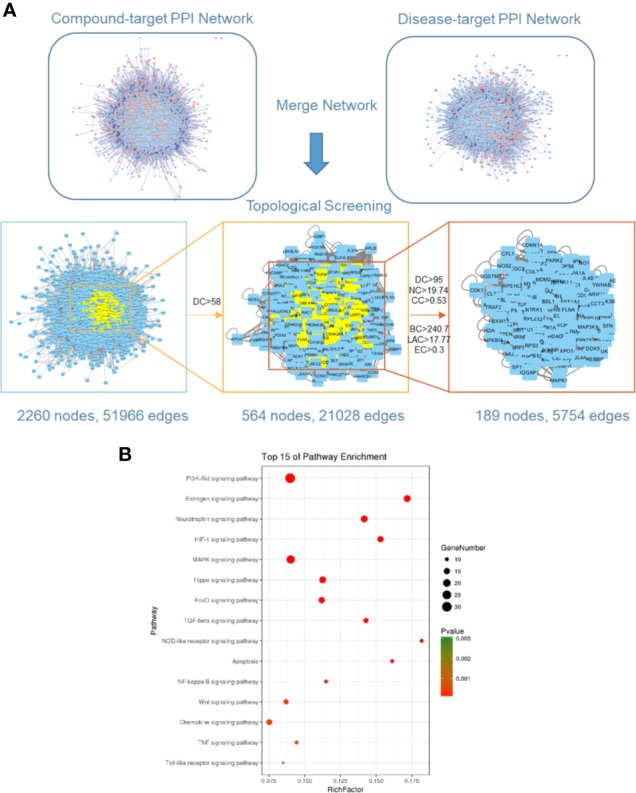
Core target identification *via* protein-protein network and Kyoto Encyclopedia of Genes and Genomes (KEGG) pathway analysis. **(A)** Topological screening of protein-protein interaction (PPI) network. **(B)** KEGG pathway analysis of core targets for RQKL in treating ischemic stroke.

Ischemic stroke-related targets are interconnected, and the PPI network has been shown to organize all protein-coding genes into a large network, which provides a better understanding of the role of various proteins in complex diseases such as ischemic stroke [29, 30]. Therefore, a compound-related target network (6,605 nodes and 158,668 edges) and a known ischemic stroke-related target network (2,861 nodes and 57,981 edges) were constructed using the PPI data. Further, we intersected both networks, consisting of 2,260 nodes and 51,966 edges. We identified nodes with degrees that were more than twice the median degree (58) of all nodes as significant targets as previously described. Thus, we constructed a network of significant targets for RQKL against ischemic stroke that had 564 nodes and 21,028 edges. Further, we calculated the topological features of each hub namely, “degree centrality (DC),” “betweenness centrality (BC),” “closeness centrality (CC),” “eigenvector centrality (EC),” “network centrality (NC),” and “local average connectivity (LAC).” The median values of “DC,” “BC,” “CC,” “EC,” “NC,” and “LAC” were 95, 240.7, 0.53, 0.3, 19.74, and 17.77, respectively. A flowchart of the core target screening is presented in **Figure 4A**. Detailed topological features of the core PPI and 189 core targets are shown in **Supplementary Table S3**.

### Kyoto Encyclopedia of Genes and Genomes Enrichment Analysis of Core Targets for Refined Qingkailing Against Ischemic Stroke

To further clarify the possible roles of the 189 core targets, we performed an enrichment analysis of their KEGG pathways. Specifically, we obtained signaling pathways related to PI3K-Akt, estrogen, neurotrophin, HIF-1, MAPK, Hippo, FoxO, TGF-beta, NOD-like receptor, apoptosis, NF-kappa B, Wnt, chemokine, TNF, Toll-like receptor, shown in **Figure 4B**. Based on the p-values, the PI3K-Akt signaling pathway was the most probable candidate for RQKL function targeting ischemic stroke.

### Experimental Validation

#### Refined Qingkailing Treatment Attenuated Ischemia-Reperfusion Injury

**Figure 5:**
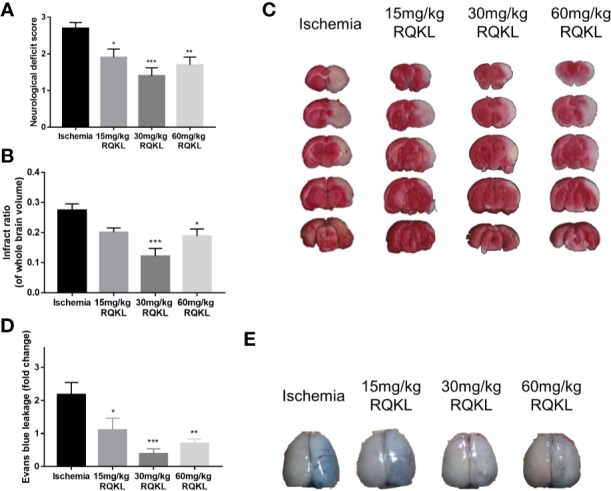
Effects of different doses of refined Qingkailing (RQKL) on ischemia/reperfusion injury. **(A)** Effects RQKL on neurological deficits score **(B)** Quantitative analysis of cerebral infarct areas. **(C)** Representative pictures of brain sections stained with 2% TTC. **(D)** Evans blue (EB) leakage analysis to evaluate prevention of blood-brain barrier (BBB) damage by RQKL. **(E)** Representative pictures of EB leakage. Data points indicate means±SEM. *vs.* ischemia group: *p<0.05, **p<0.01, ***p<0.001. At least three independent experiments were performed for each group.

To determine whether RQKL treatment improved neurological function after ischemic stroke, neurological deficit scoring was carried out. As shown in **Figure 5A**, obvious neurological deficits were observed in the ischemic group. Furthermore, while 15 mg/kg RQKL reduced the neurological deficits without a significant difference, treatment with 30 and 60 mg/kg remarkably decreased the neurological deficit score.

The infarct volume rate was assessed using TTC staining, and normal tissues were stained red, whereas the infarction area was unstained (white, **Figure 5C**). As shown in **Figure 5B**, after the 24 h tMCAO, the infarct ratio of the 30 and the 60 mg/kg groups declined obviously compared with that of the ischemic group. No significant neuroprotective effect was observed at the lowest dose.

After determining that RQKL inhibits cell death after I/R, we further investigated whether it affects the BBB integrity. In **Figures 5D, E**, the quantitative data showed that RQKL significantly decreased Evans blue extravasation leakage in the ipsilateral cortex, indicating that RQKL reduced the BBB permeability after ischemic stroke. Dose of 30 mg/kg RQKL was choose for further biological experiment because of its optimal effect.

#### Refined Qingkailing Treatment Decreased Glia Activation After Ischemic Stroke

**Figure 6:**
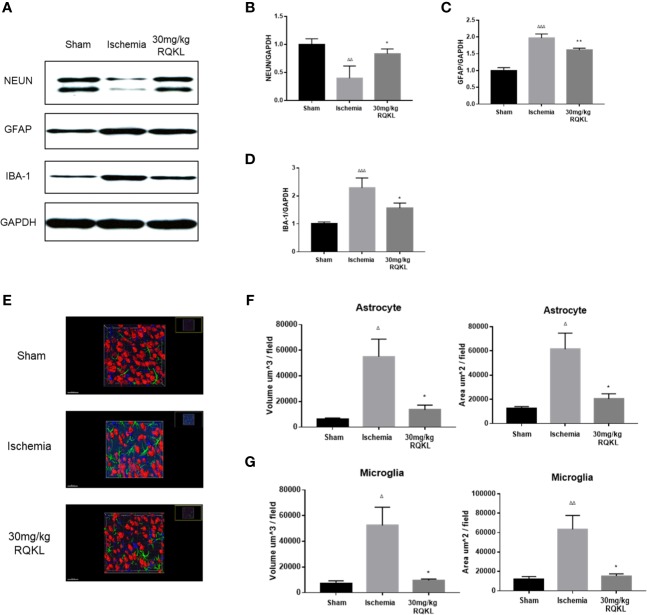
Anti-gliacyte response effect of refined Qingkailing (RQKL) on ischemia/reperfusion injury. **(A)** Representative pictures of expression of NUEN, GFAP, and IBA-1. **(B)** Quantitative analysis of NEUN expression. **(C)** Quantitative analysis of GFAP expression. **(D)** Quantitative analysis of IBA-1 expression. **(E)** Representative pictures of 3D reconstruction of astrocytes, microglia and neuron nuclei. Green represented astrocytes labeled by GFAP. Blue represented microglia labeled by INA-1. Red represented neuron nuclei labeled by NEUN. **(F)** Volume and surface area of astrocytes. **(G)** Volume and surface area of microglia. Data points indicate means±SEM. *vs.* Sham group: ^△^p < 0.05, ^△△^p < 0.01, ^△△△^p < 0.001, *vs.* ischemia group: *p < 0.05, **p < 0.01. At least three independent experiments were performed for each group.

To evaluate RQKL on neuron, astrocyte, and microglia, we evaluated expression level of protein marker of neuron, astrocyte, and microglia *via* western blot. As shown in **Figures 6A–D**, RQKL increased the expression level of NEUN compared to ischemia group, indicating a neuroprotective effect of QKL against ischemic stroke. And RQKL decreased expression level of GFAP and IBA-1 compared to ischemia group, indicating RQKL inhibited activation of astrocyte and microglia. Next we discussed RQKL on morphology of astrocyte and microglia. After 3D reconstructing astrocyte and microglia *via* software, the volume and surface area were calculated automatically. As shown in **Figures 6E–G**, ischemic stroke significantly increased the volume and surface area of astrocyte and microglia, and RQKL significantly decreased above index compared to ischemia group. These data suggested RQKL decreased glia activation after ischemic stroke.

#### Refined Qingkailing Treatment Inhibited Cell Apoptosis Induced by Ischemic Stroke

**Figure 7:**
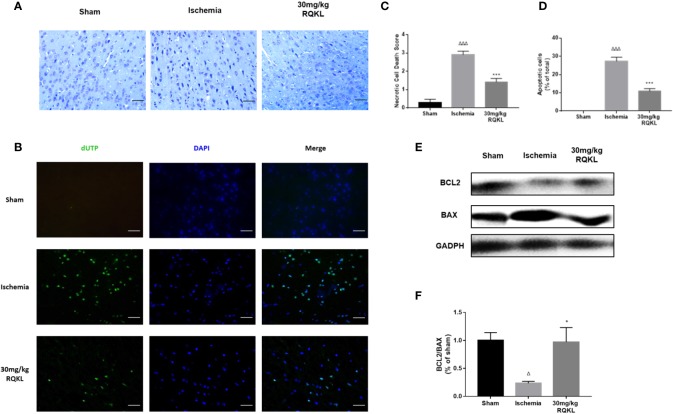
Anti-apoptosis effect of refined Qingkailing (RQKL) on ischemia/reperfusion injury. **(A)** Representative pictures of Nissl staining and **(B)** representative images of cell apoptosis stained by terminal deoxynucleotidyl transferase-mediated deoxyuridine triphosphate nick-end labeling TUNEL method. **(C)** Relevant quantitative analysis based on pathological four-score scale and **(D)** rate of apoptotic cells in each group. **(E)** Western blot analysis of B-cell lymphoma 2 (BCL2) and BCL2-associated X protein (BAX). **(F)** Quantitative analysis of BCL2: BAX expression ratio. Data points indicate means±SEM. *vs.* Sham group: ^△^p < 0.05, ^△△△^p < 0.001, *vs.* ischemia group: *p <0.05, ***p < 0.001. At least three independent experiments were performed for each group.

Integrative pharmacology showed RQKL neuroprotective effect involved in cell apoptosis signal. In the present work, Nissl staining of the cerebral cortex (**Figures 7A, C**) was used to assess the cellular protective effect of treatment with the medium-dose RQKL (30 mg/kg). The results showed that RQKL significantly decreased the necrotic cell death score based on a published four-point scale evaluating necrotic neurons in the infarct area. The TUNEL staining (**Figures 7B, D**) showed no apparent positively stained cells in the cerebral cortices of the sham group, whereas numerous apoptotic cells with positively stained nuclei were observed in the ischemic group, which also showed shrinkage of the nuclei or chromatin margination. RQKL treatment induced the percentage of apoptotic cells compared to ischemia group.

Moreover, the analysis of alterations in apoptosis-related proteins showed that RQKL dramatically increased the Bcl-2/Bax ratio in ischemic animals (**Figures 7E, F**). Thus, the overall effect of RQKL in ischemic stroke was likely mediated by both the elevation of anti-apoptotic signaling and suppression of cellular death progress.

#### Refined Qingkailing Treatment Activated PI3K/AKT Signaling Pathways

**Figure 8:**
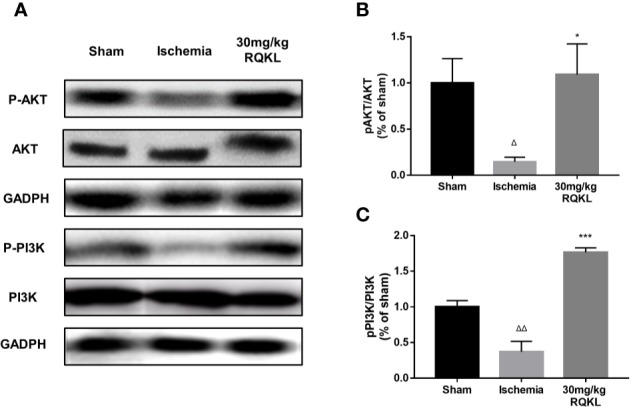
Refined Qingkailing (RQKL) activated phosphoinositide 3-kinase (PI3K)/AKT signal pathway. **(A–C)** Western blot and quantitative analysis of phosphorylated AKT (p-AKT) and p-PI3K by normalization to AKT and PI3K levels, respectively. Data points indicate means±SEM. *vs.* Sham group: ^△^p < 0.05, ^△△^p < 0.01, *vs.* ischemia group: *p < 0.05, ***p < 0.001. At least three independent experiments were performed for each group.

Previous integrative pharmacology analysis showed that the PI3K/Akt signaling pathway is the main signaling pathway mediating the biological effects of RQKL on ischemic stroke. Therefore, we designed a western blot experiment to detect the activation of this signaling pathway. As shown in **Figures 8A–C**, the results of the western blotting showed that the expression levels of p-AKT/AKT ratio and p-PI3K/ PI3K ratio in the ischemic group were significantly decreased 24 h after ischemic stroke. Moreover, RQKL significantly increased the level of the two, indicating RQKL activated PI3k/Akt signaling suppressed by ischemic stroke.

#### Refined Qingkailing Treatment Reduced Inflammatory Response

**Figure 9:**
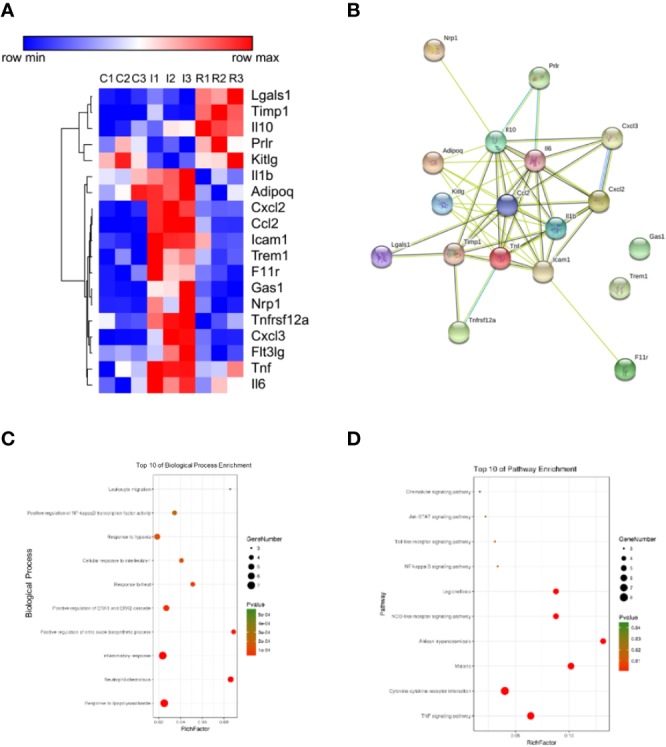
Anti-inflammation effect of refined Qingkailing (RQKL) on ischemia/reperfusion injury. **(A)** Different expression genes (DEGs) based on antibody array. **(B)** protein-protein interaction network of DEGs. **(C)** biological process enrichment of DEGs. **(D)** Kyoto Encyclopedia of Genes and Genomes (KEGG) pathway enrichment of DEGs.

To evaluate RQKL on inflammatory response, an antibody microarray containing 67 cytokines and chemokines was used. As shown in **Figures 9A–D**, 19 proteins were identified as different expression proteins of RQKL treatment compared to ischemia group. A PPI network was constructed, and BP and KEGG enrichment analysis was illustrated in bubble plots. Some BP and KEGG signals mentioned in integrative pharmacology part were obtained, including inflammatory response, response to hypoxia, TNF signaling pathway, NOD-like receptor signaling pathway, NF-kappa B signaling pathway, Toll-like receptor signaling pathway.

## Discussion

QKL injection is a famous traditional Chinese medicine formulation that has been widely used in China for more than 30 years. However, some adverse drug reactions and events have been reported, which has limited the clinical use of QKL injection since 2001 (Hao et al., 2010). Therefore, the development of a considerably safer and more efficacious QKL injection is urgently required. Therefore, we refined QKL into a novel medicine, RQKL, also targeted at treating acute ischemic stroke, one of the indications of QKL. RQKL is composed of only four main components of QKL and has the advantage of quality stabilization and clinical safety. In this study, we evaluated the neuroprotective effect of RQKL in a tMCAO rat model. We observed not only the effect of RQKL in improving neurological deficits and decreasing infract ratio but also on the BBB permeability following BBB injury in a dose-independent manner. And our unpublished *in vitro* data showed that RQKL protected neurons from ischemia/reperfusion injury with a dosage-independent manner, which was consistent with this study. And we also tested the efficacy of ingredients in RQKL against ischemia/reperfusion injury, only geniposide was found to have dose-dependent manner.

Most natural compounds are known to affect more than one gene or protein and have the potential to potently affect the entire biological process without severe side effects or excessive inhibition of certain single signaling pathway. Unfortunately, traditional methods have failed to reveal the obscure mechanisms of multi-target medicines in the systemic analysis. Recently, integrative pharmacology has provided an efficient approach for determining the underlying molecular mechanisms of multi-target medicine. We applied a integrative pharmacology approach that included target prediction, PPI analysis, GO enrichment analysis, network analysis, and experimental verification to decipher the mechanisms of the combination of four bioactive components derived from QKL injection (RQKL) for the therapy of ischemic stroke.

In this work, we built a network of 652 compound-target interactions generated between the four compounds and 438 non-repetitive targets, and numerous targets located in the cerebral cortex, hippocampus, and blood vessels, indicating a pharmacological efficacy of RQKL in treating cerebral vascular disease. GO analysis showed the multi-effects of RQKL including response to hypoxia, as well as negative regulation of cell death and inflammatory responses. Based on single cell RNA-seq data, we found RQKL could influence different cell-type, including neuron, astrocyte and microglia. Experimental data suggested an increased level of NEUN in penumbra of cortex, indicating a neuroprotective effect of RQKL treatment. According to previous studies, after ischemic stroke, astrocytes was activated into hypertrophic morphology with extended processes and swollen cell bodies and microglia was activated into hypertrophic morphology with thickened and retracted processes (Tian et al., 2019; Zhang et al., 2019). In the present study, we used 3D reconstruction software to reveal the morphology of astrocyte and microglia. We found RQKL treatment altered hypertrophic morphology of astrocyte and microglia in ischemic stroke model, indicating RQKL inhibited glia cells activation. Crucially, it is not clear whether the structural alteration of glia cells, is the cause or the consequence of protection of neurons in ischemic stroke. Further studies should focus on potential molecular mechanisms on modulating microgliosis and astrogliosis of RQKL.

After merging the PPI network of ischemic stroke- and RQKL-related targets, 189 candidate targets of RQKL treatment were characterized based on topological features. Enrichment analysis of their KEGG pathways highlighted the PI3K/Akt signaling pathway as the most probable candidate mediating the effects of RQKL on ischemic stroke. According to previous studies, suppression of PI3k/Akt signaling has been associated with the neuronal death induced by cerebral I/R. Therefore, enhanced Akt activity contributes to the anti-apoptotic and neuroprotective effects on the ischemic brain (Sun et al., 2018). Experiments showed that RQKL treatment activated PI3K/Akt signaling pathway and inhibited apoptosis progress. There are number literature reports of the cellular protective effect of compounds in RQKL *via* PI3K/Akt signal. Baicalin was reported to alleviate the neuronal apoptosis induced by ketamine toxicity (Zuo et al., 2016) and protect neonatal brains against hypoxic-ischemic injury *via* activation of PI3K/Akt signaling pathway (Zhou et al., 2017). A recent study showed that baicalin might directly bind to the pleckstrin homology domain of AKT and activate the phosphorylation of AKT on Ser473 site (Yang et al., 2019). Geniposide was reported to protect against hypoxic-ischemia-induced brain injury through the activation of PI3K/Akt signaling pathway (Liu et al., 2019). And the anti-apoptotic effects of geniposide mediated *via* the PI3K/AKT signaling pathway were also reported in H9c2 cells in response to the H/R injury (Jiang et al., 2016; Huang et al., 2017). Furthermore, numerous kinds of bile acids have been reported to activate the PI3k/Akt signaling pathway and attenuate certain kinds of brain injury (Hanafi et al., 2016; Sun et al., 2017). Activation of TGR5, a receptor of cholic acid and hyodeoxycholic acid, phosphorylate AKT at Ser473 and activate PI3K/Akt signal (Li et al., 2020). Consistent with the observation of these studies, our data showed that RQKL promoted the phosphorylation of PI3K/AKT and upregulated the ratio of the anti-apoptotic protein Bcl2 and pro-apoptotic protein Bax, which was confirmed by the downstream phosphorylation of Akt (Huang et al., 2015; Chen et al., 2017).

Our data confirmed some inflammation related signal was regulated by RQKL treatment, including TNF signaling pathway, NOD-like receptor signaling pathway, NF-kappa B signaling pathway, and Toll-like receptor signaling pathway. Ischemic stroke leads to the release of danger-associated molecular patterns (DAMPs) from dying neurons and other damaged cells. These molecules trigger the activation of resident microglia and astrocytes and leads to secretion of chemokines and cytokines, formation of an inflammatory environment, and damage amplification (Xiong et al., 2019). Modulating these inflammatory signals is a promising area of therapeutics research against ischemic stroke.

## Conclusion

In conclusion, in this study, we combined a method of Big data discovery with biological validation to study the mechanism of the actions of RQKL against ischemic stroke at the systemic level. We confirmed the neuroprotective effect of RQKL against cerebral I/R injury, which was associated with its attenuation of brain damage, cell apoptosis and activation of glia cells, modulation of the PI3K/Akt pathway, TNF signaling pathway, NOD-like receptor signaling pathway, NF-kappa B signaling pathway, and Toll-like receptor signaling pathway. Whether other pathways or mechanisms predicted in this network pharmacological approach participate in the beneficial effects of RQKL needs to be further investigated.

However, this study has some limitations that are worth mentioning. First, the reliability of the drug- and disease-related targets and protein-protein interaction database was of great importance in analyzing the underlying mechanisms of the effects of RQKL against ischemic stroke. The accuracy of GO and pathway analyses depends on *a priori* biological knowledge. Secondly, different data mining machines lead to different results. Therefore, biological verification is necessary to evaluate the reliability of bioinformatic analysis *in silico*. Lastly, more specific evidence should be considered in determining the exact effect of these drugs on this signaling pathway. Furthermore, quantitative analysis of the synergistic effect of the four compounds should be investigated in the future.

## Data Availability Statement

All datasets generated for this study are included in the article/**Supplementary Material**.

## Ethics Statement

The animal study was reviewed and approved. The animal experimental design and protocols used in this study were approved by the Ethics Review Committee for Animal Experimentation of Beijing University of Chinese Medicine (BUCM-4-2017090116-3016).

## Author Contributions

CM, XW, and TX wrote and modified the manuscript. FC and QW designed the experiment and provided fund support. CM, TX, SZ, and CZ helped to complete the animal experiment. SL carried out the western blot analysis. ZW, JM, and CL helped to construct the illustration and revised the manuscript.

## Funding

The present study was supported by grants from the National Natural Science Foundation of China (81973789, 81430102, 81774122, 81774030, 81373886, and 81303260).

## Conflict of Interest

The authors declare that the research was conducted in the absence of any commercial or financial relationships that could be construed as a potential conflict of interest.
